# Lecture on Parturient Hemorrhage

**Published:** 1860-10

**Authors:** T. Gillard Thomas

**Affiliations:** Physician to Bellevue Hospital


### LECTURE ON PARTURIENT HEMORRHAGE.

BEING THE FIFTH OF A COURSE ON

### THE COMPLICATIONS AND SEQUELÆ OF LABOR.

Delivered in the University Medical College, New York,

BY T. GILLARD THOMAS, M. D.,

Physician to Bellevue Hospital.

*Gentlemen:*—There are three distinct periods at which the child-bearing woman is liable to an inordinate loss of blood, namely, during pregnancy, during labor, and for one month subsequent to that process.

This division is by no means an arbitrary one, but is demanded by the circumstances of the case, and required for convenience of study and lucidness of understanding. Even the limit of one month given to the third variety is based upon good grounds, for at the end of that time the heretofore hypertrophied uterus having undergone involution so far as to have arrived at nearly its non-pregnant state, any flow taking place thereafter is properly regarded as disconnected with the peurperal condition.

These three distinct periods divide puerperal hemorrhage into three equally distinct varieties, viz.:

Hemorrhage before labor.
Hemorrhage during labor.
Hemorrhage after labor; or, as they may be styled for convenience of reference,

* Ante-partum hemorrhage.
Parturient "
Post-partum "

The nature of the course which now occupies us will preclude the investigation of the first of these varieties, and we will proceed at once to that of the second.

Parturient hemorrhage includes every excessive flow occurring during the act of parturition, whatever be its source, its violence, or its results.

*Sources of Parturient Hemorrhage.*

The sources from which this hemorrhage may occur are these:

(*a*) The ruptured vessels of the os and cervix uteri.

(*b*) " " " " body of the uterus.

(*c*) " " " " umbilical cord.

(*d*) " " " " vulva.

(*e*) " " " which unite the uterus and placenta.

(*a*) As the os and cervix uteri dilate in the first stage of labor, the arterioles which thickly stud the mucous membrane generally rupture, a small amount of blood pours forth, mingles with the tenacious secretion of the glands of the Naboth, and constitutes what has been called, in the language of the lying-in room, the "shew." Sometimes this flow amounts to two or three ounces, but this is exceptional, the rule being that it is just sufficient to thoroughly tinge the mucus with which it mingles. It therefore does not deserve the technical name of hemorrhage, and scarcely ever, we may even say never (unless injury has been done by the introduction of the hand or of instruments,) will it do more than alarm a primiparous woman

---

* The strict meaning of "ante-partum" is "before having brought forth," and a more exact term would be "ante-parturient," but the connexion will prevent any confusion, and the substitution of a new for an old familiar name is not desirable.

and call for an assurance of the fact just stated, on the part of the physician.

(*b*) One of the symptoms of rupture of the uterus is a free escape of blood; but recall the terrible symptoms which mark that appalling accident, and you will see at a glance that they will at once remove the case from the classification of hemorrhage, and place it in that of the most fatal of the complications of labor. In other words, the gravity of the accompanying symptoms will mask this one entirely, and cast it completely into the shade.

(*c*) Rupture of the vessels, or of one vessel, of the funis umbilicalis can at this day be no longer a matter of doubt, since in evidence of its occurrence appear the names of Delamotte, Levret, Baudelocque, Naegle, Cazeaux, and many others. It is, however, a rare accident, fortunately for diagnosis, since there are no means other than mere absence of constitutional signs on the part of the woman, by which it could be differentiated from rupture of the utero-placental vessels.

(*d*) When the flow occurs from rupture of the vessels of the vagina or bulbi vestibuli, it will generally have been the result of some violence, and our attention will likely be drawn to it by the sensation of pain on the part of the patient. Should it not, an examination, digital or ocular, will readily reveal it.

The first of the four causes which have been so far examined into is insufficient to produce a flow really deserving of the denomination of hemorrhage; the second is accompanied by other grave symptoms which make this one a secondary matter; the third and fourth are of very rare occurrence, and it may be safely announced as a rule that *whenever, during labor, a hemorrhage occurs, it arises from partial separation of the placenta from the uterus, and consequent rupture of the utero-placental vessels.*

*Varieties.*—Generally the placenta is so placed in the uterus that the os may dilate and the child be expelled without its separation being involved in these processes, and it will, under such circumstances, retain its position and the integrity of its attachment, unless some untoward accident, such as a blow or fall, occur to displace it. At other times, however, it is attached to one side of the cervix, or over the entire cervix, so as to prevent the dilatation of this part, through which the child cannot pass as long as it remains closed. Now as the os and cervix *must* be dilated to permit the passage of the child, and as their dilatation *must*, under these circumstances, to a greater or less extent, detach the placenta and rupture the utero-placental vessels, it follows, as a deduction, that hemorrhage thence resulting is not produced by accident, but, *ex necessitate rei*, is unavoidable.

For these reasons, all hemorrhages occurring during labor, have been very properly divided into

1st. Accidental hemorrhage.

2nd. Unavoidable hemorrhage.

The second variety, you perceive, is synonymous with placenta prævia, an appallation which defines the unfortunate location of the afterbirth which produces it.

Leaving the subject of placenta prævia and its resulting unavoidable hemorrhage for our next lecture, I will occupy you to-day with the consideration of that variety which is purely the effect of some accident, and which, like every other accident, might, under favorable circumstances, have been avoided.

### *Accidental Parturient Hemorrhage.*

*Frequency and Prognosis.*—You will, I imagine, get a much more correct notion of the frequency of accidental hemorrhage, by an examination of the reports of one faithful observer, than by averaging a large number of cases collected in the loose and unreliable manner which ordinarily characterizes the accumulation of statistical evidence. Dr. Collins, during a mastership of the Dublin Lying-in-Asylum of seven years, had 16,654 births occur under his supervision, and in this immense number only thirteen cases of this variety of flooding were met with; considerably less than one in one thousand. Small as this proportion is, however, it is larger than it should be for true accidental parturient hemorrhage, since Dr. Collins brought under the same head all those cases occurring during the three last months of pregnancy and during labor.

Of the thirteen women thus attacked, two died, and both after serious operations : one after version, and the other after craniotomy, so that it is by no means proper to conclude that they died from the hemorrhage. Of the children one only was born alive.

Thus you will perceive that the accident is not of frequent occurrence, that the prognosis for the mother is good, and that that for the child is decidedly bad. I refrain from giving you other statistical statements on this point, from the fact that authors generally confound the two first varieties of hemorrhage together.

*Causes.*—The pathological state causing the flow, is, as already mentioned, rupture of the vessels which pass from the uterus into the placenta. The causes which bring about such rupture are numerous, since any kind of violence sufficiently great for the separation of the placenta would accomplish it.

The chief are—Blows or falls.
Sudden uterine contraction from mental emotion.
Sudden shocks or successions given to the uterus, as from laughter, vomiting, &c.
Dragging off of the placenta by shortness of the cord, or its repeated winding around the child's neck.
Placental apoplexy occurring near the periphery of the organ.

There are other and less frequent and conspicuous causes, but it would be useless to name them, since, as I have said, any accident which severs the utero-placental attachment would produce it.

*Symptoms and Diagnosis.*—As the prognosis, and more especially the treatment of the two varieties of parturient hemorrhage differ from each other very much indeed, it is of great importance that the accoucheur should determine at once as to which one he has to deal with, and that his decision be as far as possible positive and final. This he will in many cases do without difficulty, but sometimes he will have to remain in suspense for a short period until the progress of the case enlightens him and determines the point.

Denman on this point justly remarks: "Before there is some dilatation of the os uteri, be the discharge ever so profuse, and it may even at this time be excessive, I do not know that it is always possible to tell with certainty whether the placenta is present or not. It may indeed be conjectured that the placenta is there attached by the cushion-like feel of the cervix and lower parts of the uterus." He then goes on to remark how, even after dilatation of the os, a clot of blood may be mistaken for the placenta.

The only reliable means for determining the nature of the flow are these:

*In Accidental Hemorrhage,*

[*a*] There will have been no ante-partum loss.
[*b*] Uterine efforts will diminish the flow.
[*c*] An evident cause will generally be found for it.
[*d*] The loss is not generally very profuse.
[*e*] The placenta cannot be touched.
[*f*] Os uteri will be natural to the touch.
[*g*] Placental murmur loudest near fundus.

*In Unavoidable Hemorrhage,*

[*a*] There will have been hemorrhage during the last month or months of pregnancy.
[*b*] Uterine efforts will increase the flow.

[*c*] No cause will be found for it.
[*d*] The loss is often sudden and profuse.
[*e*] The edge of the placenta may be touched.
[*f*] Os uteri will be thicker than ordinary.
[*g*] Placental murmur loudest in one or other iliac fossa.

As a little reflection will readily explain to you why these two varieties should be characterized by their respective symptoms, I will not do more thah enunciate them. Let me insist, however, upon the importance of an early and positive diagnosis, if such is within the range of possibility. Of all the symptoms mentioned, the presence of the placenta near the os is the most valuable, and this one you must thoroughly test. Do not be satisfied with temporizing with digital examinations if they are not sufficient, but explaining the necessity to your patient, pass the entire hand into the vagina; if the os is dilatable pass the index finger well up into the cervical canal, and ascertain to your full satisfaction whether you have or have not a case of placenta prævia to deal with. As a matter of course, if the rational signs point strongly to the supposition that the case is one of accidental hemorrhage, and there is no immediate danger, you would not expose your patient to the annoyance and pain attendant upon this procedure; but far better would it be to err on that side, than by a culpable inactivity to remain ignorant of a point upon the knowledge of which so much will depend.

*Treatment*–A parturient uterine hemorrhage shouid be treated upon precisely the same principles which should guide us in the management of such an accident taking place from any other part of the body. This you may, at first thought, regard as a sweeping assertion; but as we proceed you will perceive that, although from the nature of the locality from which the flow occurs, the means employed for *developing* the principles may differ, the *principles* themselves are identical.

Let us suppose, for example, that a hemorrhage should occur from any part of the surface of the body, as the result of a wound or abrasion, and let us follow out the principles which one after another would be employed by the surgeon, until he finally succeeds in checking it.

1st. If the flow were slight the patient would be kept perfectly quiet, and an effort made to constringe the mouths of the bleeding vessels by cold and styptic applications, as ice, alum, tannin, matico, etc.

2nd. Should these very useful and commonly employed hæmostatic agents fail in making this principle effective, an attempt might be made to cause in the wound the formation of a coagulum, which, extending up into the mouths of the bleeding vessels, might seal them up as is done by plugging the anterior nares alone, or with the posterior, in epistaxis.

3rd. Should this fail, a very excellent principle, that of closing the open arterioles by firmly compressing their walls, might be developed by direct pressure, as is done, for instance, in hemorrhage from the palmar arch, by placing a billiard ball in the palm of the hand, and binding it firmly in its place by a bandage.

4th. Should even this fail, still another and surer one exists in the application of a ligature to the bleeding vessels; and to it the surgeon would now with confidence resort.

Thus, one after another he has brought to his assistance four principles, each valuable in itself, each differing from the one tried before it, and all capped by one which is as certain in its results as human means can ever be.

Thus, too, gentlemen, in parturient hemorrhage the obstetrician should act; and he will find that, if the first three of these four principles fail him, he, like the surgeon, will have one left which will prove as certain as the ligature.

In establishing these principles always be mindful of the pathological state which causes the dangerous symptoms which they are to control; i. e. that a portion of the placenta has been torn off from its uterine attachment, and that from its disrupted face, as well as from the corresponding surface of the uterus from which it was torn, the blood is welling forth.

In a case of accidental parturient hemorrhage, the first indication to be fulfilled is to check the flow by constringing the mouths of these vessels; and this will best be accomplished by confining the patient to bed in the supine posture, and absolutely prohibiting all muscular effort or mental exercise, even that attendant upon speaking; by keeping the apartment cool; by administering cold, acidulated drinks, as lemonade, or water acidulated with the elixir of vitriol; by applying towels soaked with cold water, or vinegar and water, to the vulva and over the uterus, and by prescribing astringents, as tannic or gallic acid in full dose, which being carried to the bleeding vessels by the circulation, may aid in producing the same result which their local application effects in vascular rupture elsewhere.

If by these means we succeed, we will have good cause for congratulation, for we will have relieved the woman without having in any way sacrificed the chances of her child. If they do not succeed, then we must resort to some other plan which may prove more effectual, and we enter into the consideration of the adoption of the second principle. The only available means at our command for causing a clot to form in utero, under these circumstances, is the tampon or vaginal plug, an agent advised by many, and one which might accomplish the result as perfectly as do the double tampons employed in epistaxis. But there are dangers attending its use so great, that I must not only guard you against them, but advise an avoidance of this means in parturient hemorrhage, except in rare and particular cases. I would say in advance, *avoid the tampon as a rule, after the seventh month of pregnancy, but employ it boldly, even at full term, in a few exceptional and peculiar cases.*

The tampon, gentlemen, may be styled one of the most useful and dangerous of our uterine hæmostatics, and it is really curious to see how different and even contradictory is the advice which is given concerning the propriety of its employment. Let me, by an excusable and called-for digression, endeavor to fix in your minds this morning a few maxims concerning it.

A plug introduced into the vagina, of sufficient size to fill the canal, acts in uterine hemorrhage in these two ways—preventing the escape of the fluid which is flowing out of the uterus; this collects, and gradually is "backed" into the cavity above; soon it distends this cavity to its utmost extent; if the fœtal mass is present, insinuates itself between the chorion and uterine wall, and at last forcibly dilating the os by distension of the whole organ, produces a powerful expulsive effort which frequently expells child, accumulated blood, and tampon together. When the uterus is not dilatable by the accumulating blood, this fluid coagulates within its cavity; the coagulum beginning to form at the os, extends upwards towards the source of the hemorrhage, and will often seal up the mouths of the bleeding vessels.

Both these results are often very desirable, and to accomplish them no means compares with the tampon. But after the seventh month of pregnancy the uterus is so large that it may contain a sufficient amount of blood to produce death, so that from this period to the completion of labor it is always attended by danger. (I need not insist upon the gross impropriety of the employment of such a means after delivery.)

Thus then, although the tampon might effect much for us in parturient hemorrhage, as a rule it should not be employed; and, in exceptional cases which demand it, should be resorted to only after mature consideration, and its effects be watched with very careful scrutiny. Observe these rules in using it.

Never employ the tampon *from choice* when there is a possibility of a dangerous internal hemorrhage.

At full term do not employ it after the waters have been discharged, for then the uterus will accommodate a large amount of blood.

Never emyloy it at full term after your patient has lost a great deal of blood, or from natural feebleness of body would be endangered by even a slight hemorrhage.

In a strong woman who has not already lost a good deal of blood, in whom the uterus is contracting well, and whose bag of waters has not been ruptured, I would not hesitate to employ it if other means failed, or from any reason I deemed them inapplicable.

Should the principle which is developed by the tampon be beyond our reach on account of the danger of the means which accomplished it, or, should it have been unsuccessfully resorted to, how are we to avail ourselves of the third?

You remember that the flow of blood in accidental parturient hemorrhage is checked by uterine contraction, and that this is so marked as to constitute one of its characteristic symptoms; now let us examine this fact. When the organ contracts, the bleeding surfaces of the placenta and uterus are pressed firmly against the fœtal mass, and thus their open vessels are shut. If we could cause this pressure to be continuous and powerful, at the same time that it was resisted by a hard mass, we would cause the flow to cease entirely, and would be acting exactly as the surgeon does who binds the billiard ball in the palm of the hand. But you may ask how are we to introduce a hard resisting body into the uterus to act as counterpart of the ivory ball? We are supplied with such a substance in the body of the child. Surrounded by the soft and pliable bag of waters, one chief object of which is to prevent its hardness from being perceived by the sensitive uterus, it lacks the feature of resistance which we now desire; but evacuate the surrounding waters by puncturing the bag, and instantly the unyielding body presses against the hemorrhagic spot, and the principle is developed.

This, however, although often sufficient, is not always so, the pressure not being powerful enough. Under such circumstances, in the case of a palmar hemorrhage, the surgeon would remove his loose bandage, and apply another which would make all the pressure desirable. And so the obstetrician, by the administration of small doses of ergot, can so force his point of resistance against the bleeding surface as to compress entirely the ruptured vessels and render them impermeable. By these means you not only bring to your aid the principle mentioned, but, to a certain extent, you will establish that which will be mentioned fourth, for the vessels are diminished by the same contractions which press the child against the bleeding surface. According to my experience it is rare for them to fail. In fact, I have never known them to do so in true accidental hemorrhage. Should they do so, however, but one resort remains, and that is ligation of the vessels from which the obstinate current flows. Have we any means by which ligatures may be thus applied in utero? Again bountiful nature comes to our aid, and we have but to use the means which she presents us and our end is accomplished. After every natural labor, were there not some arrangements for checking the flow from the broken utero-placental vessels, a hemorrhage would occur, but so soon as the uterus is emptied the fibres contract, diminish its size very greatly, and being arranged around the mouths of the vessels as the meshes of a netted purse are around the finger which is pushed through them, they inevitably close the mouths, and prevent all sanguineous loss.

After having tried in vain, by the development of the three principles mentioned, to accomplish what we wish, naught remains but to empty the uterus, force it into contraction, and cause nature to do what the surgeon does in external hemorrhage. If the head can be seized by the forceps, employ them; should it be out of their reach, accept of version as the alternative, and deliver promptly. Thus by successive steps the scientific obstetrician advances from mild, harmless, but correspondingly inefficient means, to more dangerous, and proportionately more effectual ones, until he arrives at a point at which he can safely say, "I will by this surely succeed in staying the flow, and will rescue my patient from its dangers."

But do not despise the more inefficient means because a more effectual one exists. Would you not blame the surgeon, who, for a slight hemorrhage, should tie the supplying arteries without seeing what might be done by styptics, pressure, etc.?

Keep the most efficient agent in reserve, *because it is accompanied by danger for mother and child*, and always strive to accomplish your ends by the mildest, least dangerous, and apparently most trifling means. Should you succeed, a host of unthought-of evils lurking like harpies in the shade, may by your moderation be avoided; should you not, then promptly apply the most efficient and most dangerous of your resources, which, like a "corps de reserve" you have kept until the fitting moment.

What has been denominated, gentlemen, "heroic practice," often marks the course of the ignorant and unreflecting obstetrician ; and although the vulgar may be blinded by its show of energy, decision, and promptness, and led to believe it an evidence of knowledge, it will often bring about consequences alike disastrous and avoidable.

The skilful general does not fire a twelve-pounder at a handful of marauders who could be dispersed by a musket-shot, nor does he trust to his muskets when an army is upon him in its might.

Never lose sight, too, of this fact in treating a complication of labor, that the interests of two beings are intrusted to your care, and that while you are to do all in your power for those of the mother, those of the child are scarcely less imperative. If, then, in the treatment of this accident, you can adopt means which will accomplish both ends, give them by all means the preference over those which, even if more surely effectual, in removing the woman from danger, will sacrifice the chances of the child.

The older one grows in obstetric experience, the more convinced does he become that many a woman has died from the unnecessary introduction of the hand into the uterus ; that many a uterus has been ruptured by uncalled for violence ; and that Herod destroyed not a tithe of the children which have been killed in utero by the reckless use of ergot.

The following is a resume of the treatment which has been recommended in this lecture, the principle upon which each procedure acts being italicised.

1st. *Constringe the bleeding vessels* by cold to the uteru sand vulva, acidulated drinks, astringents, and perfect rest in the recumbent posture.

2d. In cases of failure *cause a clot to form in the mouth of the bleeding vessels* by the tampon, should the case be one in which this practice would be safe.

3d. Should this fail, *make direct pressure against the bleeding vessels* by evacuation of the waters, and increase it if necessary by the use of ergot.

4th. None of these means succeeding, *ligate the vessels* by evacuating the uterus, and causing firm contraction.

As I have alluded to certain cases in which the tampon might, in a woman for whom we did not fear a slight loss of blood, be preferable to an immediate resort to rupture of the membranes, it may be well for me to give you an example. There are several cases where it might be preferable, but this will serve as a type : in a transverse presentation before the os is dilatable, rupture of the bag and administration of ergot would much complicate the operation of version, and thus endanger both mother and child. Should accidental hemorrhage occur in such a case, then it would be advisable to gain time for dilatation of the os by the use of a means which offers the probability of checking the flow without wasting the precious fluid which is to facilitate a dangerous operation.

Because this means is attended by danger I would not entirely discard it; but let that be a sufficient reason for its not being employed, except when absolutely necessary, and for its effects being watched with the utmost caution.—*American Medical Times.*

